# Selection and Validation of Novel Biomarkers for ntOPN‐Based Models for Diabetic Kidney Disease in Patients With Diabetes Mellitus

**DOI:** 10.1155/jdr/8209388

**Published:** 2026-07-18

**Authors:** Lu-Xi Zou, Zhi-Li Hou, Chen-huan Qian, Xue Wang, Ling Sun

**Affiliations:** ^1^ School of Management, Xuzhou Medical University, Xuzhou, Jiangsu, China, xzmc.edu.cn; ^2^ Department of Nephrology, Xuzhou Clinical School of Xuzhou Medical University, Xuzhou, Jiangsu, China, xzmc.edu.cn; ^3^ Department of Nephrology, Xuzhou Central Hospital, Southeast University, Xuzhou, Jiangsu, China, seu.edu.bd

**Keywords:** diabetic kidney disease, growth differentiation factor 15 (GDF15), nomogram, n-terminal osteopontin (ntOPN), prediction model

## Abstract

**Introduction:**

We previously found that urinary n‐terminal osteopontin (ntOPN) performed well for predicting diabetic kidney disease (DKD). This study is aimed at screening potential biomarkers for improving ntOPN‐based models in DKD detection and prediction.

**Methods:**

We performed a cross‐sectional and then prospective cohort study. The novel biomarkers for DKD development were selected by the SOMAscan platform. The selected biomarkers were further validated by the SHapley Additive exPlanations (SHAP) algorithm, Pearson correlation, and logistic regression. The ntOPN‐based models for DKD prediction were established, evaluated, and utilized by machine learning.

**Results:**

The baseline growth differentiation factor 15 (GDF15) was selected by SOMAscan assays, and urinary GDF15 was validated as an independent predictor for DKD occurrence (adjusted OR 1.43, 95% CI 1.20–1.75) and progression (adjusted OR 1.39, 95% CI 1.15–1.75) by multivariate logistic regression. The receiver operating characteristic (ROC) analysis showed that the multibiomarker panel consisting of urinary ntOPN‐to‐creatinine ratio (UntOCR) and urinary GDF15‐to‐creatinine ratio (UGCR) had stronger abilities in forecasting the 2‐year risk of DKD occurrence (AUC 0.838 vs. 0.818) and DKD progression (AUC 0.867 vs. 0.834) than the combination of estimated glomerular filtration rate (eGFR_cr-cys_) and urinary albumin‐to‐creatinine ratio (UACR). A nomogram was further built with a high C‐index (0.8433).

**Conclusions:**

Compared with eGFR_cr-cys_ combined with UACR, the models based on urinary ntOPN and GDF15 could provide more accurate tools for DKD prediction. Our attempt might provide a feasible approach for searching promising biomarkers for clinical applications.

## 1. Introduction

Diabetic kidney disease (DKD) has emerged as the predominant cause of chronic kidney disease (CKD) globally [1]. DKD may result in rapid progression to end‐stage renal disease (ESRD), high cardiovascular risk, as well as substantial healthcare expenditures [2]. Early prediction of DKD could be crucial for improving cardiac and renal prognosis, along with lowering healthcare expenses for DM patients.

DKD is a clinical diagnosis mainly based on persistent albuminuria and elevated serum creatinine [3]. However, albuminuria is not specific for DKD; besides, around 20% of patients with Type 1 DM (T1DM) and 40% of Type 2 DM (T2DM) patients remain normoalbuminuric until their estimated glomerular filtration rate (eGFR) <60 mL/min/1.73m^2^, which is defined as nonproteinuric DKD [4]. Serum creatinine is not sensitive to true GFR decline, as it merely begins to increase after nephron loss > 50% [5]. Therefore, elevated albumin‐to‐creatinine ratio (UACR) and reduced eGFR are the final consequences of DKD, and they should be regarded as late indicators for DKD screening. Renal biopsy is considered the gold standard, although it is invasive and is usually performed in DM patients with manifestations of other kidney diseases [3]. More promising markers for identifying early DKD are urgently needed.

Osteopontin (OPN) has been reported to be involved in multiple pathophysiological pathways, such as cell adhesion, migration, and survival, bone turnover, inflammation, and immunity [6]. OPN is mainly expressed in distal nephrons and the loop of Henle in normal kidneys, and is significantly upregulated in glomeruli and all segments of the tubule after renal damage [7]. OPN structurally contains two terminal zones, the C‐terminal and N‐terminal. The N‐terminal osteopontin (ntOPN), containing integrin receptor binding domains, has a stronger profibrotic adhesion effect than full‐length OPN, and plays an important role in mediating cell adhesion and inflammation [8]. We previously reported that urinary N‐terminal osteopontin (UntOPN) was associated with DKD development and performed better than the traditional clinical biomarkers of serum creatinine combined with UACR in predicting DKD [9].

Screening novel proteins that contribute to early DKD development may reveal DKD pathophysiology and improve the accuracy of DKD diagnosis and prediction. Previous studies had performed proteomics assays to detect early DKD [10], and distinguish DKD patients with high risk of ESRD progression [11]. Few studies reported the early detection of patients with a high risk of DKD in a DM population without renal injury. This study is aimed at building a novel strategy for screening biomarkers in DKD development using the SOMAscan platform, validate the effect of selected biomarkers on detecting patients with DKD high risk, and optimize ntOPN‐based forecast models for renal outcomes in patients with DM.

## 2. Methods

### 2.1. Study Design and Participants

This is a cross‐sectional and then prospective cohort study. During the cross‐sectional phase, DM patients were recruited according to the following inclusion criteria: (1) T1DM ≥ 5 years (all patients with T1DM received insulin therapy), or T2DM (Table S1) [12, 13]; (2) age ≥18 years old; and exclusion criteria: (1) without complete clinical data; (2) had verified nondiabetic kidney diseases (non‐DKD), major cardiovascular and cerebrovascular disorders, tumors, systemic diseases, infections, or malnutrition at first clinic attendance; (3) women during pregnancy or lactation; (4) refused to give informed consent.

During the subsequent prospective phase, further exclusion criteria were applied to select DM patients from the cross‐sectional cohort: (1) had verified DKD at baseline [3]; (2) had persistent albuminuria, and/or decreased renal function at first clinic attendance; (3) without complete data of 2‐year follow‐up (Figure S1). The study was approved by the Ethics Committee at Xuzhou Central Hospital (Reference Number XZXY‐LJ‐20201030‐055).

### 2.2. Proteomics Assay

Baseline plasma samples underwent proteomic analysis targeting 11,000 proteins by SomaScan Platform (AccuraMed, Shanghai). A slow off‐rate modified aptamer‐based assay was performed using specific affinity‐binding reagents (SOMAmers) and a SomaScan microarray (SomaLogic, Inc.).

### 2.3. Enzyme‐Linked Immunosorbent Assay (ELISA)

Baseline plasma and urine samples underwent competitive ELISA, the ntOPN and selected protein growth differentiation factor 15 (GDF15) (Cat. JL20845 and JL19786, Jianglaibio, China), with the coefficient of variation (CV) < 10% in both inter‐ and intra‐assay precision analyses. Urine creatinine was measured to correct the protein quantification values, which were calculated as the urine protein‐to‐creatinine ratio.

### 2.4. Clinical Outcomes for the Prospective Cohort

GFR estimated by the CKD‐EPI equation was recorded as eGFR_cr-cys_ using serum creatinine and cystatin‐C [14], and recorded as eGFR_cr_ using serum creatinine [15]. The primary endpoint was the DKD occurrence, defined as having a causal relationship between DM history and UACR/eGFR changes, excluding other glomerular diseases and systemic diseases, and with the presence of one of the following conditions [3]: (1) repeat UACR ≥ 30 mg/g at least 2 of 3 measurements within 3–6 months; (2) eGFR < 60 mL/min/1.73m^2^ for more than 3 months; (3) renal pathological findings corroborated the diagnosis of DKD.

The secondary endpoints were defined as “DKD progression,” which included one of the following conditions: (1) sustained decrease in eGFR by at least 25%; (2) progression to ESRD, and/or need for renal replacement therapy; and (3) death from renal cause.

### 2.5. Sample Size Estimation for the Prospective Cohort

The sample size was estimated using the following formula:

n=Z2×p1−pδ2.

Using a two‐sided significance level of 5%, the corresponding Z value was 1.96. The expected population proportion *p*, was assumed to be 0.50, and the acceptable margin of error, *δ* was set at 0.10. The minimum sample size was 96. Following the adjustment for a design effect ranging from 1 to 3 and the allowance for an anticipated 10% loss to follow‐up, the required number of participants was estimated to range from 107 to 320.

### 2.6. Data Collection and Measurements

Baseline socio‐demographic and clinical data were documented, including age, gender, body mass index (BMI), comorbidities, medications, and laboratory parameters. BMI = weight (kg)/(Height [m])^2^, measured using a calibrated height and weight scale (Cat. RGZ‐120, Jiangsu Suhong Medical Equipment, China). Fasting blood and first‐morning void urine samples were obtained for clinical routine indicators and novel biomarkers detection. Clinical laboratory measurements were made in the certified laboratory of Xuzhou Central Hospital. The plasma and urine samples were centrifuged at 3500 rpm for 10 min at 4°C and stored at −80°C until biomarkers were assayed.

### 2.7. Statistical Analysis

For SomaScan proteomics assays, we performed volcano plots to show the differential expressions of proteins between groups, and performed protein–protein interaction (PPI) networks to explore the vital role of selected proteins in DKD pathophysiology using the STRING Database. The artificial intelligence SHapley Additive exPlanations (SHAP) algorithm was employed to elucidate the predictive ability of random forest models and rank the importance of variables in determining clinical outcomes. Spearman′s rank correlation was employed to evaluate the interdependence among variables. Univariate and multivariable logistic regression analyses were performed to analyze the effects of baseline biomarkers on predicting the 2‐year risk of DKD. Receiver operating characteristic (ROC) analysis was employed to assess the predictive models for clinical outcomes. The predictive performance of the established models was assessed using two crucial metrics: the integrated discrimination improvement (IDI) and net reclassification improvement (NRI). A nomogram model was constructed to achieve clinical application. Statistical significance was set at a two‐sided *p* value < 0.05. All analyses were conducted using SAS software (Version 9.4, SAS Institute Inc., United States) and R software (Version 4.2.2, https://www.r-project.org).

## 3. Results

### 3.1. Cohort Characteristics and Plasma Proteomics Assays

We recruited 134 DM patients without baseline kidney injury in the prospective cohort. Of these, 13 patients had DKD progression, and 83 patients had no renal involvement during the follow‐up. Because of the high cost of SOMAscan measurements, we randomly selected baseline plasma samples from nine patients with “DKD progression” (9/13) and 5 patients without renal involvement (5/81) as “control group” for proteomics assays (Figure S1).

In comparison with the control group, the patients with DKD progression were older (*p* = 0.014), had longer duration of DM (*p* = 0.009) and higher baseline UACR (*p* = 0.003). No differences were observed between the two groups concerning the remaining variables at baseline. After 2‐year follow‐up, the patients with DKD progression had significantly higher levels of UACR and serum cystatin C, along with lower levels of eGFRcr and eGFRcr‐cys. (Table 1).

**Table 1:** Baseline and 2‐year follow‐up characteristics of the patients in proteomics assays.

Variable	Baseline				2‐year follow‐up			
	Total	Control group	DKD progression	p	Total	Control group	DKD progression	*p*
Number	*N* = 14	*N* = 5	*N* = 9		*N* = 14	*N* = 5	*N* = 9	
Age	53.5 [50.2; 60.8]	50.0 [32.0; 51.0]	60.0 [54.0; 73.0]	0.014	54.5 [51.2; 61.8]	51.0 [33.0; 52.0]	61.0 [55.0; 74.0]	0.014
Female, *n* (%)^a^	5 (35.7%)	3 (60.0%)	2 (22.2%)	0.266	5 (35.7%)	3 (60.0%)	2 (22.2%)	0.266
Duration of diabetes (years)	8.00 [2.00; 18.8]	1.00 [0.00; 1.00]	18.0 [7.00; 21.0]	0.009	9.00 [2.00; 19.8]	2.00 [1.00; 2.00]	19.0 [8.00; 22.0]	0.009
UACR (mg/g)^b^	16.8 [10.3; 23.8]	7.00 [6.20; 9.60]	23.0 [17.0; 24.2]	0.003	22.0 [8.35; 52.1]	6.50 [6.10; 7.00]	40.9 [27.3; 56.3]	0.003
Albumin (g/L)	42.2 [40.2; 45.3]	42.3 [42.2; 46.2]	41.9 [39.8; 42.7]	0.257	42.2 [39.5; 45.8]	46.2 [44.4; 47.2]	41.9 [36.6; 42.2]	0.053
Prealbumin (mg/L)	234 [177; 260]	222 [214; 255]	246 [135; 262]	0.841	250 [193; 284]	230 [181; 255]	256 [244; 287]	0.463
Cystatin C (mg/L)	0.81 [0.77; 0.98]	0.78 [0.77; 0.79]	0.90 [0.80; 1.02]	0.062	0.94 [0.70; 1.02]	0.70 [0.69; 0.70]	1.01 [0.95; 1.19]	0.014
UA (*μ*mol/L)	311 [281; 380]	382 [325; 390]	286 [258; 348]	0.109	342 [241; 426]	316 [264; 432]	365 [234; 409]	0.947
Creatinine (*μ*mol/L)	43.8 [31.7; 53.7]	48.4 [46.5; 57.0]	38.6 [30.0; 47.3]	0.257	55.1 [48.1; 83.0]	48.4 [46.5; 55.0]	79.8 [53.6; 96.1]	0.125
eGFRcr (mL/min/1.73m^2^)	122 [108; 132]	126 [112; 128]	119 [103; 144]	0.641	105 [71.0; 118]	122 [108; 126]	88.3 [60.7; 104]	0.014
eGFRcr‐cys (mL/min/1.73m^2^)	112 [90.1; 126]	124 [110; 127]	99.8 [84.2; 117]	0.205	95.5 [83.3; 124]	128 [114; 131]	84.9 [63.0; 93.9]	0.006
HbA1c (%)	9.70 [7.00; 10.8]	9.70 [7.00; 10.8]	9.70 [7.00; 10.9]	0.738	7.10 [6.82; 10.7]	7.00 [6.80; 10.8]	7.20 [6.90; 10.2]	0.640
TCH/HDL‐C ratio	3.83 [2.91; 4.44]	4.38 [2.80; 4.40]	3.78 [2.98; 4.45]	0.841	3.53 [2.87; 4.53]	3.04 [2.54; 3.44]	3.78 [3.28; 6.07]	0.125
APOB/APOA1 ratio	0.87 [0.61; 1.00]	0.92 [0.56; 0.95]	0.83 [0.65; 1.01]	0.641	0.63 [0.50; 0.79]	0.62 [0.48; 0.63]	0.67 [0.56; 0.80]	0.549
Hemoglobin (g/L)	142 [130; 150]	151 [118; 156]	142 [136; 147]	0.640	126 [116; 145]	118 [118; 138]	129 [115; 146]	0.738
NLR	1.92 [1.46; 2.42]	1.90 [1.44; 2.47]	1.95 [1.52; 2.29]	0.841	2.00 [1.39; 2.83]	2.06 [1.95; 2.88]	1.56 [1.37; 2.67]	0.463

Abbreviations: APOA1, apolipoprotein A1; APOB, apolipoprotein B; DKD, diabetic kidney disease; eGFR, estimated glomerular filtration rate; HDL‐C, high‐density lipoprotein cholesterol; NLR, neutrophil to lymphocyte ratio; TCH, total cholesterol; UA, uric acid; UACR, urinary albumin‐to‐creatinine ratio.

^a^Discrete values expressed as number (percentage).

^b^Continuous values expressed as means (SD) if normally distributed or median (interquartile range) if skewed.

**Figure 1:**
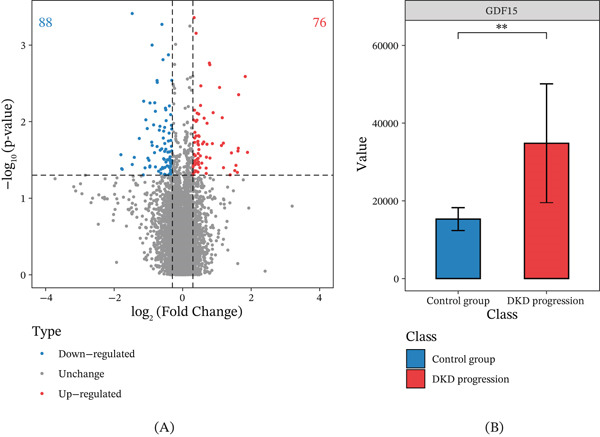
Plasma proteome profiling of the selected 14 participants. (A) A volcano plot demonstrated the differential expression of proteins between DM patients without DKD and those with DKD progression (*p* < 0.05, FC > 1.5, and FC < 1/1.5). Blue: downregulated proteins; gray: proteins that were not significantly changed; red: upregulated proteins. (B) Boxplots visually represent the dispersion of RFU values for GDF15 in plasma. GDF15, growth differentiation factor 15; RFU, relative fluorescence units. ∗∗*p* < 0.01, by multiple comparison.

SomaScan assays showed that even before DM patients present with renal involvement, there were still 76 upregulated proteins (all *p* < 0.05, fold change (FC) > 1.5), and 88 downregulated proteins (all *p* < 0.05, FC < 1/1.5) at baseline in the DKD progression group (Figure 1A). Of these upregulated proteins, the GDF15 expression showed a significant difference (*p* = 0.0036, log_2_FC = 1.08) (Figure 1B). Furthermore, GDF15 is located at the core of the PPI network, indicating that it might play a crucial role in DKD development (Figure S2).

### 3.2. Validity of GDF15 for DKD Diagnosis in the Cross‐Sectional Cohort

The UntOPN has been proven to be an independent predictor for DKD occurrence and progression [9]. To investigate the ability of GDF15 to detect DKD from DM patients, the plasma and urinary GDF15 were measured together with ntOPN. In the cross‐sectional cohort, we enrolled 316 DM patients, among whom 95 patients had verified DKD (Figure S1, Table S2). The patients with DKD had higher levels of urinary GDF15 and ntOPN, plasma GDF15, lipoprotein a, TCH/HDL‐C, APOB/APOA1, and neutrophil‐to‐lymphocyte ratio (NLR), and lower levels of hemoglobin than the patients without renal involvement (Table S2).

**Figure 2:**
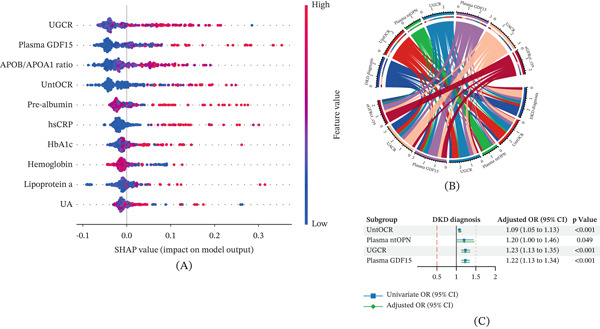
Performance of GDF15 in DKD detection from DM patients. (A) Shapley values of variable importance for DKD diagnosis. Each point on the graph is a Shapley feature for each individual; its position on the *y*‐axis was determined by the importance of variable, and its location on the *x*‐axis was determined by its Shapley value. For each variable, the Shapley values for each patient were plotted horizontally, with its magnitude represented by the *x*‐axis. The importance of the variables was determined by their total Shapley values across all patients. The colors denoted different Shapley values from low to high, with low values in blue and high values in red. For example, as the plasma GDF15 increased (from blue to red), the Shapley values increased, indicating that the likelihood of DKD diagnosis increased. In this SHAP algorithm, the UGCR and plasma GDF15 ranked in the top two, indicating that both circulating and urinary GDF15 levels were strongly associated with DKD development. (B) Pearson correlation coefficients among GDF15, ntOPN, and DKD diagnosis. The width of the line represented the values of the correlation coefficients. (C) Forest plots of GDF15, ntOPN for DKD diagnosis based on logistic regression. Blue squares represent the OR values of the univariate regression analysis, and green diamonds represent the OR values of the multivariate logistic regression adjusted by age, gender, and BMI. DKD, diabetic kidney disease; DM, diabetes mellitus; BMI, body mass index; UGCR, urinary growth differentiation factor 15 to creatinine ratio; GDF15, growth differentiation factor 15; APOA1, apolipoprotein A1; APOB, apolipoprotein B; UntOCR, urinary n‐terminal osteopontin to creatinine ratio; ntOPN, urinary n‐terminal osteopontin; hsCRP, high‐sensitive C‐reactive protein; UA, uric acid; UACR, urinary albumin to creatinine ratio; eGFR, estimated glomerular filtration rate; OR, odds ratio; CI, confidence interval.

To identify variables contributing most to DKD detection, their Shapley values displayed the Top 10 contributors impacting DKD diagnosis, except for traditional variables in DKD criteria, such as UACR, eGFR, and serum creatinine (Figure 2A). The high values of plasma GDF15 were clustered in the positive Shapley values, indicating that high urinary and plasma GDF15 contributed to positive DKD diagnosis. For one patient with a high Shapley value (0.96), the high urinary growth differentiation factor 15 to creatinine ratio (UGCR) (839 ng/mmol), plasma GDF15 (291 pg/mL), urinary N‐terminal osteopontin‐to‐creatinine ratio (UntOCR) (88 *μ*g/mmol), and ApoB/ApoA1 ratio (1.71) were the main sources of DKD risk (Figure S3).

Spearman correlation showed that plasma GDF15, UGCR, and UntOCR were positively associated with DKD diagnosis (*R* = 0.2706, 0.2654, and 0.2695, respectively, all *p* < 0.001) (Figure 2B, Table S5). The multivariate logistic regression demonstrated that elevated GDF15 and ntOPN in plasma and urine all increased the risk of DKD diagnosis, adjusted by age, gender, and BMI (Figure 2C).

### 3.3. Validity of ntOPN and GDF15 for DKD Prediction in the Prospective Cohort

For the prospective cohort, a total of 134 DM patients without baseline kidney injury were recruited, among whom eight patients developed non‐DKD, and 45 patients developed DKD during the 2‐year follow‐up (Figure S1). Compared with patients without DKD, the patients with DKD onset had higher baseline levels of UntOCR, UGCR, and UACR, indicating that even if UACR levels were within the normal range, their absolute values could still predict the risk of DKD occurrence (Table S3).

Similarly, compared with patients without DKD progression, the patients with DKD progression had higher baseline levels of UntOCR, UGCR, and UACR, with lower serum creatinine and higher eGFR_cr_, which revealed that renal hyperfiltration could be the earliest hemodynamic abnormality of DKD (Table S4).

#### 3.3.1. Variable Importance for Clinical Outcomes Using Shapley Values

**Figure 3:**
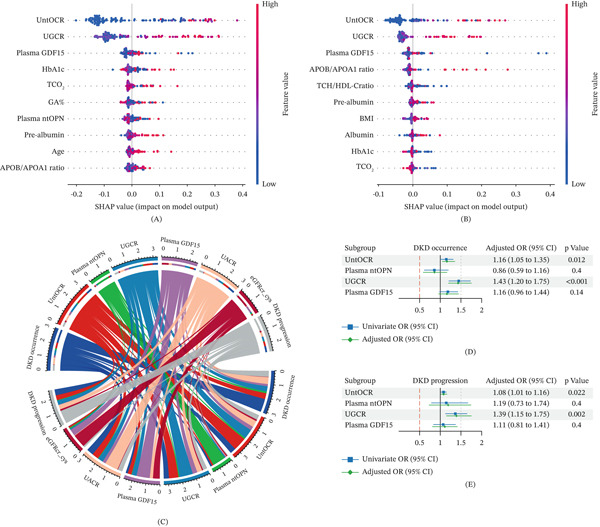
Performance of GDF15 in DKD prediction in DM patients. (A) Shapley values of variable importance for DKD occurrence. (B) Shapley values of variable importance for DKD progression. (C) Pearson correlation coefficients among GDF15, ntOPN, DKD occurrence, and progression. The width of the line represented the values of the correlation coefficients. (D) Forest plots of the levels of GDF15, ntOPN for DKD occurrence based on logistic regression. (E) Forest plots of the levels of GDF15, ntOPN for DKD progression based on logistic regression. Blue squares represent the OR values for the univariate regression analysis, and green diamonds represent the OR values for the multivariate logistic regression adjusted by age, gender, and BMI. DKD, diabetic kidney disease; DM, diabetes mellitus; BMI, body mass index; UntOCR, urinary n‐terminal osteopontin‐to‐creatinine ratio; ntOPN, urinary n‐terminal osteopontin; UGCR, urinary growth differentiation factor 15‐to‐creatinine ratio; GDF15, growth differentiation factor 15; UACR, urinary albumin‐to‐creatinine ratio; TCO_2_, total carbon dioxide; GA, glycosylated albumin; eGFR, estimated glomerular filtration rate; TCH, total cholesterol; HDL‐C, high‐density lipoprotein cholesterol; APOA1, apolipoprotein A1; APOB, apolipoprotein B; OR, odds ratio; CI, confidence interval.

To evaluate the performance of variables that contributed to DKD prediction, the SHAP graph displayed the Top 10 variables for DKD occurrence (Figure 3A) and DKD progression (Figure 3B) using random forest models. Both graphs showed that UntOCR, UGCR, and plasma GDF15 ranked Top 3 and contributed to the likelihood of clinical outcomes, indicating that both ntOPN and GDF15 could be promising predictors for DKD occurrence and progression in DM patients. To better interpret the risks on the patient level, we also performed the individual‐level risk predictions by the Shapley values using the random forest models (Figures S4 and S5).

#### 3.3.2. ntOPN and GDF15, Novel Biomarkers for DKD Prediction

Spearman correlation showed that both baseline levels of UntOCR and UGCR were positively associated with DKD occurrence (*R* = 0.4406 and 0.3948, respectively, all *p* < 0.001), and DKD progression (*R* = 0.3640 and 0.3199, respectively, all *p* < 0.001) (Figure 3C and Table S6). Moreover, the high levels of UntOCR and UGCR significantly increased the risk of DKD occurrence and progression, after the adjustment of age, gender, and BMI by the multivariate logistic regression, which indicated that both ntOPN and GDF15 were independent risk factors for DKD development (Figure 3D,E).

#### 3.3.3. Performance of ntOPN‐Based Models for DKD Prediction

**Figure 4:**
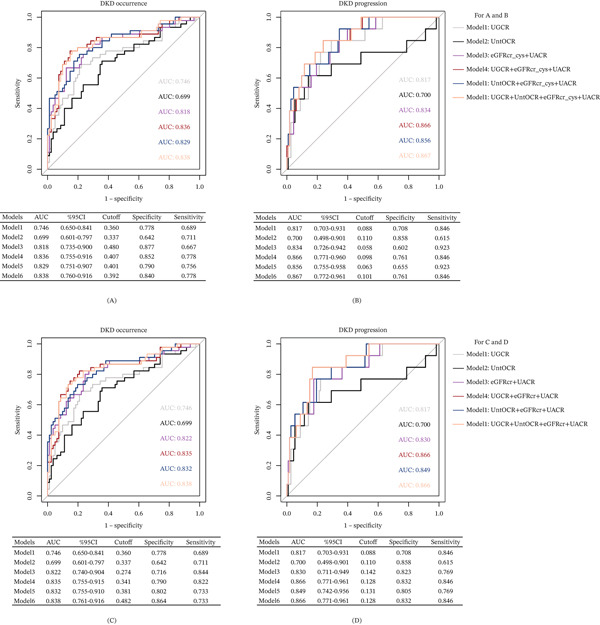
The receiver operating characteristic (ROC) curves of models for DKD prediction are adjusted by age, gender, and BMI. (A) Prediction models for 2‐year risk of DKD occurrence in the prospective cohort using eGFR_cr-cys_. The AUC values ranked from high to low as follows: the four‐biomarker model consisted of UGCR, UntOCR, eGFR_cr-cys_, and UACR (orange), then UGCR + eGFR_cr−cys_ + UACR (red), UntOCR + eGFR_cr−cys_ + UACR (blue), eGFR_cr−cys_ + UACR (purple), UGCR (gray), and UntOCR (black). (B) Prediction models for 2‐year risk of DKD progression in the prospective cohort using eGFR_cr-cys_. (C) Prediction models for 2‐year risk of DKD occurrence in the prospective cohort using eGFR_cr_. (D) Prediction models for 2‐year risk of DKD progression in the prospective cohort using eGFR_cr_. The AUC and its 95% CI, cutoff value, specificity, and sensitivity were listed below the ROC curves for each model. DKD, diabetic kidney disease; ROC, receiving operating characteristic; BMI, body mass index; UGCR, urinary growth differentiation factor 15 to creatinine ratio; UntOCR, urinary n‐terminal osteopontin to creatinine ratio; eGFR, estimated glomerular filtration rate; UACR, urinary albumin to creatinine ratio; AUC, area under the curve; CI, confidence interval.

To explore the ability of ntOPN and GDF15 to predict the 2‐year risk of DKD, a series of biomarker panels were established. The four‐biomarker model consisted of UGCR, UntOCR, eGFR_cr-cys_, and UACR (orange), ranked the top for predicting the 2‐year risk of DKD occurrence with the area under the curve (AUC) 0.838 and a cutoff value of 0.392 (Figure 4A). Compared with the model of eGFR_cr-cys_ + UACR, the AUC of the four‐biomarker model significantly increased from 0.818 to 0.838, with NRI and IDI estimated to be 0.558 (*p* = 0.0018) and 0.0467 (*p* = 0.0229), respectively (Table S7).

In forecasting the 2‐year risk of DKD progression, Figure 4B showed that the AUC value of UGCR + UntOCR + eGFR_cr−cys_ + UACR (orange) was also the highest among the six ROC curves. Compared with the model of eGFR_cr−cys_ + UACR, the AUC of the four‐biomarker model increased from 0.834 to 0.867, with NRI estimated to be 0.7297 (*p* = 0.007) (Table S7).

Similar results were obtained in the models that consist of UGCR, UntOCR, eGFR_cr,_ and UACR (orange) (Figure 4C,D). Comparably, GDF15‐based models (Figure S6A, B, E, and F) and the ntOPN‐based models (Figure S6C, D, G, and H) presented similar trends for forecasting DKD occurrence and DKD progression (Table S7).

#### 3.3.4. Establishing Traditional and Dynamic Nomogram Models for DKD Occurrence

**Figure 5:**
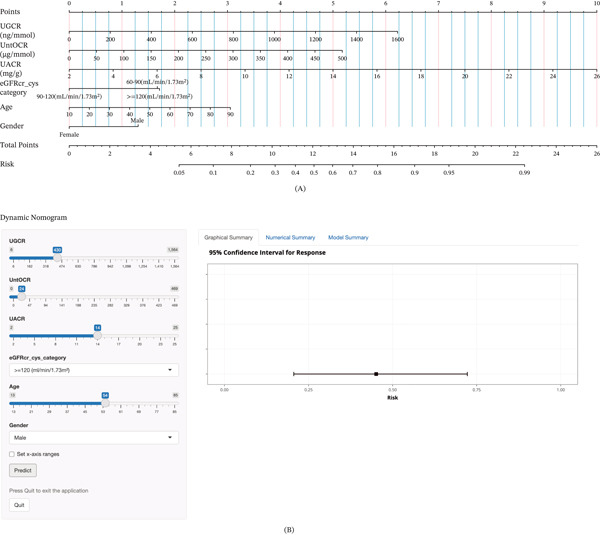
(A) Traditional nomogram to predict the 2‐year risk of DKD occurrence for DM patients. Evaluation of the probability of 2‐year risk of DKD onset needs three steps. First, the vertical lines were drawn for each predictor to obtain their points from the “Points” axis; then, the total point is the sum of all predictor points; third, the probability was calculated by the value from the “Total points” axis to the “Risk” axis; the larger value of total points, the higher risk of DKD development in DM patients. (B) Dynamic nomogram to predict the 2‐year risk of DKD occurrence for DM patients. Its steps to estimate the DKD risk: first, enter the value of each variable; second, click on the “Predict” button; then, the result will be displayed on the right side as a predictive value and its 95% CI. Abbreviations: UGCR, urinary growth differentiation factor 15‐to‐creatinine ratio; UntOCR, urinary n‐terminal osteopontin to creatinine ratio; UACR, urinary albumin to creatinine ratio; eGFR, estimated glomerular filtration rate; OR, odds ratio; CI, confidence interval.

The importance of variables for predicting the 2‐year risk of DKD onset was calculated and ranked by the SHAP method; the top two independent predictors, together with traditional DKD biomarkers of eGFR_cr-cys_ and UACR, were selected to build a traditional nomogram (Figure 5A). The C index of our nomogram was 0.8433, suggesting a good predictive accuracy for DKD occurrence. For instance, a 60‐year‐old (point = 1.9) male (point = 1.3) DM patient with normal UACR (20 mg/g) (point = 7.5), normal eGFR (120 mL/min/1.73m^2^) (point = 1.75), high levels of UGCR (800 ng/mmol) (point = 3.1) and UntOCR (100 *μ*g/mmol) (point = 1.1) would have a total of 16.7 points, which suggested that the probability of DKD onset was about 87% in the following 2 years. Furthermore, a dynamic nomogram was generated to enhance the usability of the model in clinical practice (Figure 5B).

## 4. Discussion

Globally, DKD has emerged as the primary contributor to ESRD, with a 5‐year survival rate below 65.7% among ESRD patients [16], whereas the annual medical costs in China approached US $15,000 per ESRD patient [17]. A previously validated decision model for cost‐effective lifetime strategies for the management of DKD demonstrated that the screening strategy (early detection of DKD followed by renin‐angiotensin system [RAS] inhibitors) was more cost‐effective with superior clinical outcomes than the control strategy (no intervention) in patients with T2DM [18]. The DKD onset is usually insidious; proactive screening models for DKD may prove beneficial in terms of renal prognosis in patients with DM.

Nicholas et al. were the first to demonstrate that full‐length OPN was critical in DKD progression in experimental model animals of T1DM and T2DM [19], possibly through modulation of renal inflammation, oxidative stress, and fibrosis [20, 21]. Then, OPN was reported to contribute to aggravating DKD and all‐cause mortality in DM patients [22]. The ntOPN has a stronger profibrotic adhesion effect than full‐length OPN [8]. However, the association between ntOPN and DKD development was unclear until we reported that urinary ntOPN was associated with DKD development, which could be a promising marker for DKD prediction [9]. This study demonstrated that ntOPN could be a promising predictor for DKD diagnosis, occurrence, and progression (SHAP algorithm, Figures 2 and 3). Moreover, we planned to further discover novel biomarkers to enhance the ability of the ntOPN‐based models in forecasting DKD development.

GDF15, also known as macrophage inhibitory cytokine‐1 (MIC‐1), belonging to the transforming growth factor‐*β* (TGF‐*β*) superfamily, is a stress‐inducing cytokine with anti‐inflammatory, antifibrotic, and antiproliferative properties [23]. Plasma and urinary GDF15 likely reflect distinct (though potentially overlapping) pathophysiological processes. Mechanistically, plasma GDF15 arises from systemic multiorgan stress responses, whereas urinary GDF15 originates from both filtered plasma GDF15 and local renal production—the latter potentially indicating renal pathophysiological changes. Previous studies reported that elevated serum GDF15 could be associated with old age (≥ 65 years old), smoking, cardiometabolic risk, incident CKD, and rapid decline of renal function [24]. Furthermore, elevated circulating GDF15 has been implicated in the development of T2DM‐associated complications, particularly DKD [25]. Consistently, urinary GDF15 levels were found to be significantly increased in patients with histologically diagnostic DKD compared with DM patients without DKD [26]. In addition, GDF15 was demonstrated to reduce food intake and body weight. GDF15 elevation might have a protective effect against DM deterioration, and its agonists might provide new targets for DM management [27]. However, few studies discussed the expression of GDF15 in DM patients before DKD development, and the role of GDF15 in detecting patients with high risks of DKD progression remains unknown.

SOMAscan proteomics analysis showed that plasma GDF15 was significantly elevated in the DM patients who would develop progressive DKD in the next 2 years, and PPI network demonstrated its core position in the upcoming DKD progression. The plasma and urinary GDF15 were measured in a larger DM population to verify the predictive role of GDF15 in DKD diagnosis, occurrence, and progression. Our results demonstrated that GDF15 could be a promising biomarker for DKD prediction. Moreover, urinary GDF15 could outperform ntOPN in predicting the 2‐year risk of DKD occurrence and progression, with larger values of AUC (Figure 4).

The DKD development is the result of multiple pathophysiological processes [28], therefore, the multibiomarker panels should enhance the predictive capacity. Based on the above findings, combined with traditional indicators, the predictive models based on ntOPN and/or GDF15 were constructed for 2‐year risk of DKD development in DM patients, which performed better than eGFR_cr-cys_ combined with UACR. The ntOPN‐based models combined with GDF15 present the largest AUC (Figure 4 and S6, Table S7). In addition, a nomogram consisting of six variables (UGCR, UntOCR, UACR, eGFR_cr-cys_, age, and gender) facilitated rapid calculation of the 2‐year risk of DKD occurrence in clinical practice, with visual advantages and a high C‐index. Its high C‐index indicated that this model could be accurately and widely applied in the DM population.

Risk factors affecting DKD could be categorized as susceptibility factors (e.g., age, sex, and family history), initiation factors (e.g., acute kidney injury and hyperglycemia), and progression factors (e.g., hypertension, dyslipidemia, hyperuricemia, obesity, heart problems, and lifestyle) [29]. Of which, hypertension gradually increases GFR decline and albuminuria, then accelerates DKD progression [30]. Uric acid mediates RAS activation and endothelial dysfunction, induces vascular smooth muscle proliferation, and promotes microangiopathy [31]. Long‐term exposure to hyperuricemia is associated with DKD development and progression [32]. Abnormal lipid profiles have been demonstrated to be associated with renal dysfunction in patients with DM, the elevated serum lipoprotein (a) and ApoB/ApoA1 ratio are independent predictors for incident reduced renal function [33] and DKD progression to ESRD [34]. TCH/HDL‐C ratio increases susceptibility to DM, hypertension, and CVD [35], whereas few studies reported its relationship with DKD. Besides conventional risk factors, the systemic inflammatory status was also a prominent predictor for DKD [36]. The NLR was reported to improve predicting renal function loss over traditional risk factors in T2DM patients [37]. Our previous study also proved that neutrophil percentage could contribute to constructing a nomogram for DKD prediction in T2DM patients [38].

This study observed that the comorbidity of hypertension and hyperuricemia could be potential risk factors for DKD diagnosis in the cross‐sectional cohort. However, their prevalences were not detected as predictors for DKD occurrence and progression in the prospective cohort (Table S2, S3, and S4). In terms of abnormal lipid metabolism, we observed that the higher levels of lipoprotein a, APOB/APOA1, and TCH/HDL‐C ratio were associated with DKD diagnosis (Table S2); the APOB/APOA1 ratio could be a risk factor for DKD diagnosis, occurrence, and progression; and the TCH/HDL‐C ratio could be a predictor for DKD progression (SHAP algorithm, Figures 2 and 3). The NLR (Table S2) and hsCRP (Figure 2) might be potential risk factors for DKD diagnosis, whereas no inflammatory biomarker was statistically significant in forecasting DKD occurrence and progression in the prospective cohort. All these clinical parameters could be promising markers for establishing predictive models for DKD in future studies.

This study exhibited several strengths. Firstly, few studies reported the role of ntOPN and GDF15 in early predicting DKD and its renal prognosis in DM patients. This research stood as the first endeavor to address the clinical implementation of GDF15 in establishing models to detect and predict DKD. Secondly, our study demonstrated that urinary ntOPN and GDF15 were associated with the development of DKD. We further improved the ntOPN‐based models with GDF15 for DKD prediction, which presented the largest AUC for DKD occurrence and progression. Additionally, our study attempted to screen meaningful biomarkers through proteomic assays and then validated them in a larger population using multiple machine learning algorithms, which might provide a new approach for searching more promising biomarkers for clinical applications.

This study had limitations. The latest guideline for DKD [13] recommended that the RAS inhibitors, sodium‐dependent glucose transporters 2 (SGLT‐2) inhibitors, and glucagon‐like peptide‐1 receptor agonist (GLP‐1RA) can improve the renal prognosis of DM patients. However, these drugs were underused in our enrolled participants. Therefore, these medications had no significant difference between the control group and DKD onset/progression in the prospective cohorts. This unachieved optimal management might contribute to relatively rapid DKD progression in our study. All patients with T1DM in this study received insulin therapy. Since insulin offers no renal protection beyond glycemic control and does not improve renal outcomes, its use was not included in the statistical analysis. The glycemic benefits of insulin therapy could be reflected by fasting blood glucose and HbA1c levels (Table S2, S3, and S4).

## 5. Conclusions

In conclusion, the results of this study demonstrated that urinary ntOPN and GDF15 were independent predictors for DKD occurrence and progression in DM patients. In comparison with the models of eGFR_cr-cys_ combined with UACR, our multibiomarker models based on ntOPN and GDF15 performed better in predicting DKD development. This suggests that they could provide more accurate tools for assessing DKD risk. Our research may contribute to early screening and intervention, thereby helping to prevent and delay the progression of DKD in patients with DM.

## Author Contributions

L.S.: conceptualization, methodology, data curation, formal analysis, funding acquisition, writing—original draft, writing—review and editing. X.W.: investigation. Z‐L.H.: investigation. C.Q.: investigation. L‐X.Z.: conceptualization, methodology, data curation, formal analysis, funding acquisition, writing—original draft, writing—review and editing.

## Funding

This study was supported by the Research Project of Jiangsu Provincial Commission of Health (ZD2022044); Clinical Observership in NHS Hospitals London U.K Project of Jiangsu Commission of Health, the Preventive Medicine Research Project of Jiangsu Commission of Health (Y12023008); Major Project of Philosophy and Social Science Research in Colleges and Universities of Jiangsu Province (2024SJZD062); 333 High‐level Personnel Cultivation Project in Jiangsu Province ([2022]3‐12‐151); Science and Technology Foundation of the Xuzhou Health Committee (XWKYHT20240026); and Science and Technology Foundation of Xuzhou City (KC25078).

## Disclosure

All authors have read and approved the final version of the manuscript. Corresponding author have full access to all of the data in this study and takes complete responsibility for the integrity of the data and the accuracy of the data analysis.

## Ethics Statement

This study adhered to the International Conference on Harmonization guidelines for Good Clinical Practice and was conducted in accordance with the Declaration of Helsinki. The protocol was approved by the Ethics Committee of Xuzhou Central Hospital (NO. XZXY‐LJ‐20201030‐055).

## Conflicts of Interest

The authors declare no conflicts of interest.

## Supporting information


**Supporting Information** Additional supporting information can be found online in the Supporting Information section. Table S1: Criteria for Type 1 DM and Type 2 DM. Table S2: Baseline characteristics of the enrolled 316 DM patients in the cross‐sectional cohort. Table S3: Baseline characteristics stratified by DKD onset in enrolled 126 DM patients in the prospective cohort. Table S4: Baseline characteristics stratified by DKD progression in enrolled 126 DM patients in the prospective cohort. Table S5: Spearman correlation coefficients among the novel biomarkers and DKD diagnosis in the cross‐sectional cohort. Table S6: Spearman correlation coefficients among the novel biomarkers and clinical outcomes in the prospective cohort. Table S7: The IDI and NRI for models in Figure 4 and Figure S6. Figure S1: Overview of study design and cohort participants. In the prospective cohort phase, a total of 134 DM patients without kidney injury were included and followed. Because of the high cost of SOMAscan measurements, we randomly selected baseline plasma samples from nine patients with DKD progression (red square) and five patients without renal involvement (green square) during the follow‐up period for further proteomics assays. DM, diabetes mellitus; T1DM, Type 1 diabetes mellitus; T2DM, Type 2 diabetes mellitus; DKD, diabetic kidney disease; non‐DKD, nondiabetic kidney diseases; eGFR, estimated glomerular filtration rate. Figure S2: Protein–protein interaction (PPI) network showing the interactions of differentially expressed proteins based on STRING database. The darker the node, the more core the interaction. Figure S3: Individuals with a (A) high SHAP value and (B) a low SHAP value for DKD diagnosis in the cross‐sectional cohort. Figure S4: Individuals with (A) a high SHAP value and (B) a low SHAP value for DKD occurrence in the prospective cohort. Figure S5: Individuals with (A) a high SHAP value and (B) a low SHAP value for DKD progression in the prospective cohort. Figure S6: The receiver operator characteristic (ROC) curves of models for DKD prediction, adjusted by age, gender, and BMI. The AUC and its 95% CI, cutoff value, specificity, and sensitivity were listed below the ROC curves for each model. DKD, diabetic kidney disease; BMI, body mass index; GDF15, growth differentiation factor 15; UGCR, urinary growth differentiation factor 15‐to‐creatinine ratio; ntOPN, n‐terminal osteopontin; UntOCR, urinary n‐terminal osteopontin‐to‐creatinine ratio; eGFR, estimated glomerular filtration rate; UACR, urinary albumin‐to‐creatinine ratio; AUC, area under the curve; CI, confidence interval.

## Data Availability

The data supporting the findings of this study are available from the corresponding author upon reasonable request. These data are not publicly accessible because they contain information that could compromise the privacy of research participants or are subject to ethical restrictions.
