# Supplementary material for: Selection and Validation of Novel Biomarkers for ntOPN‐Based Models for Diabetic Kidney Disease in Patients With Diabetes Mellitus
Source: J Diabetes Res. 2026 Jul 18;2026:8209388. doi: 10.1155/jdr/8209388 (PMC13380030; doi:10.1155/jdr/8209388)
Supplement: Supplementary file 1 — Supporting Information Additional supporting information can be found online in the Supporting Information section. Table S1: Criteria for Type 1 DM and Type 2 DM. Table S2: Baseline characteristics of the enrolled 316 DM patients in the cross‐sectional cohort. Table S3: Baseline characteristics stratified by DKD onset in enrolled 126 DM patients in the prospective cohort. Table S4: Baseline characteristics stratified by DKD progression in enrolled 126 DM patients in the prospective cohort. Table S5: Spearman correlation coefficients among the novel biomarkers and DKD diagnosis in the cross‐sectional cohort. Table S6: Spearman correlation coefficients among the novel biomarkers and clinical outcomes in the prospective cohort. Table S7: The IDI and NRI for models in Figure 4 and Figure S6. Figure S1: Overview of study design and cohort participants. In the prospective cohort phase, a total of 134 DM patients without kidney injury were included and followed. Because of the high cost of SOMAscan measurements, we randomly selected baseline plasma samples from nine patients with DKD progression (red square) and five patients without renal involvement (green square) during the follow‐up period for further proteomics assays. DM, diabetes mellitus; T1DM, Type 1 diabetes mellitus; T2DM, Type 2 diabetes mellitus; DKD, diabetic kidney disease; non‐DKD, nondiabetic kidney diseases; eGFR, estimated glomerular filtration rate. Figure S2: Protein–protein interaction (PPI) network showing the interactions of differentially expressed proteins based on STRING database. The darker the node, the more core the interaction. Figure S3: Individuals with a (A) high SHAP value and (B) a low SHAP value for DKD diagnosis in the cross‐sectional cohort. Figure S4: Individuals with (A) a high SHAP value and (B) a low SHAP value for DKD occurrence in the prospective cohort. Figure S5: Individuals with (A) a high SHAP value and (B) a low SHAP value for DKD progression in the prospective cohort. Fig [file JDR-2026-8209388-s001.docx]

**Table S1. Criteria for Type 1 diabetes and Type 2 diabetes[1]**

| **Step 1. Criteria for the diagnosis of diabetes in nonpregnant individuals, meet any one of the following:** |
| --- |
| - HbA1C ≥6.5% (≥48 mmol/mol). The test should be performed in a laboratory using a method that is NGSP certified and standardized to the DCCT assay.* |
| - FPG ≥126 mg/dL (≥7.0 mmol/L). Fasting is defined as no caloric intake for at least 8 h.* |
| - 2-h PG ≥200 mg/dL (≥11.1 mmol/L) during OGTT. The test should be performed as described by the WHO, using a glucose load containing the equivalent of 75 g anhydrous glucose dissolved in water.* |
| - In an individual with classic symptoms of hyperglycemia or hyperglycemic crisis, a random plasma glucose ≥200 mg/dL (≥11.1 mmol/L). Random is any time of the day without regard to time since previous meal. |
| **Step 2. Classification for Type 1 diabetes and Type 2 diabetes:** |
| - Type 1 diabetes: due to autoimmune β-cell destruction, usually leading to absolute insulin deficiency, including latent autoimmune diabetes in adults. |
| - Type 2 diabetes: due to a nonautoimmune progressive loss of adequate β-cell insulin secretion, frequently on the background of insulin resistance. |

DCCT, Diabetes Control and Complications Trial; FPG, fasting plasma glucose; OGTT, oral glucose tolerance test; NGSP, National Glycohemoglobin Standardization Program; WHO, World Health Organization; 2-h PG, 2-h plasma glucose. *In the absence of unequivocal hyperglycemia, diagnosis requires two abnormal results from different tests, which may be obtained at the same time (e.g., HbA1C and FPG), or the same test at two different time points. For patients with type 1 diabetes, insulin therapy was essential and mandatory.

Reference:

1. Amer Diabet Assoc Professional Practice Comm D: **2. Diagnosis and Classification of Diabetes: Standards of Care in Diabetes-2026**. *Diabetes Care* 2026, **49**:S27-S49.

Table S2. Baseline characteristics of the enrolled 316 DM patients the cross-sectional cohort.

|  | Total  (N=316) | DM  (N=221) | DKD  (N=95) | p.overall |
| --- | --- | --- | --- | --- |
| Sociodemographics: |  |  |  |  |
| Age | 55.0 [47.0;62.0] | 55.0 [47.0;62.0] | 56.0 [49.5;64.5] | 0.084 |
| Female, n (%)^a^ | 128 (40.5%) | 85 (38.5%) | 43 (45.3%) | 0.315 |
| BMI (kg/m^2^)^b^ | 24.5 [22.1;27.0] | 24.4 [22.1;27.1] | 24.6 [21.9;26.8] | 0.689 |
| Duration of diabetes (years) | 7.00 [2.00;13.0] | 6.00 [1.00;12.0] | 8.00 [4.00;16.0] | 0.011 |
| Smoking, n (%) | 102 (32.3%) | 73 (33.0%) | 29 (30.5%) | 0.760 |
| Alcohol, n (%) | 84 (26.6%) | 64 (29.0%) | 20 (21.1%) | 0.187 |
| Comorbid illness |  |  |  |  |
| Hypertension, n (%) | 133 (42.1%) | 79 (35.7%) | 54 (56.8%) | 0.001 |
| Hyperlipemia, n (%) | 164 (51.9%) | 112 (50.7%) | 52 (54.7%) | 0.590 |
| Hyperuricemia, n (%) | 54 (17.1%) | 26 (11.8%) | 28 (29.5%) | <0.001 |
| Medication |  |  |  |  |
| RAS inhibitor, n (%) | 66 (20.9%) | 39 (17.6%) | 27 (28.4%) | 0.044 |
| SGLT-2 inhibitor, n (%) | 102 (32.3%) | 78 (35.3%) | 24 (25.3%) | 0.106 |
| GLP-1RA, n (%) | 34 (10.8%) | 26 (11.8%) | 8 (8.42%) | 0.496 |
| Metformin, n (%) | 152 (48.1%) | 115 (52.0%) | 37 (38.9%) | 0.044 |
| Laboratory data |  |  |  |  |
| UntOPN (ng/ml) | 44.1 [25.3;118] | 38.0 [23.7;81.5] | 73.4 [35.5;507] | <0.001 |
| UntOCR (μg/mmol) | 5.03 [2.68;18.8] | 3.83 [2.51;12.9] | 11.3 [3.74;77.2] | <0.001 |
| Plasma ntOPN (ng/mL) | 168 [125;248] | 167 [129;236] | 172 [124;300] | 0.242 |
| UGDF15 (pg/mL) | 3579 [2244;4674] | 3226 [2106;4272] | 4423 [2743;5058] | <0.001 |
| UGCR (ng/mmol) | 388 [283;578] | 360 [267;478] | 527 [332;822] | <0.001 |
| Plasma GDF15 (pg/mL) | 212 [126;368] | 165 [116;288] | 291 [149;626] | <0.001 |
| UACR (mg/g) | 16.2 [9.10;32.3] | 11.3 [7.30;16.9] | 104 [38.7;300] | <0.001 |
| TCO_2_ (mmol/L) | 25.8 [23.8;27.3] | 25.9 [23.9;27.6] | 25.6 [23.6;27.1] | 0.293 |
| Albumin (g/L) | 43.5 [40.4;45.8] | 43.8 [41.2;46.2] | 42.4 [37.7;45.2] | 0.002 |
| Globulin (g/L) | 25.9 [22.8;28.7] | 26.0 [22.9;28.7] | 25.9 [22.7;28.7] | 0.904 |
| Pre-albumin (mg/L) | 242 [191;278] | 244 [203;280] | 225 [166;277] | 0.034 |
| GA% | 17.6 [13.7;25.7] | 17.2 [13.6;25.6] | 18.8 [14.7;24.8] | 0.489 |
| α-HBDH (U/L) | 140 [123;159] | 137 [121;152] | 150 [129;175] | <0.001 |
| ALP (U/L) | 89.9 [71.9;112] | 88.0 [71.2;108] | 98.9 [74.5;119] | 0.021 |
| CK (U/L) | 76.5 [49.0;101] | 76.0 [49.0;95.0] | 78.0 [50.0;104] | 0.694 |
| BUN (mmol/L) | 5.88 [4.86;7.36] | 5.87 [4.90;6.95] | 5.99 [4.69;8.46] | 0.161 |
| Cystatin C (mg/L) | 0.88 [0.79;1.05] | 0.86 [0.78;0.98] | 0.98 [0.83;1.33] | <0.001 |
| UA (μmol/L) | 287 [235;362] | 285 [233;347] | 294 [244;406] | 0.070 |
| Creatinine (μmol/L) | 47.0 [38.6;56.8] | 47.0 [38.6;55.0] | 47.8 [38.9;66.5] | 0.057 |
| eGFR_cr_ (ml/min/1.73m^2^) | 114 [102;127] | 116 [108;128] | 109 [90.5;123] | 0.001 |
| eGFR_cr-cys_ (ml/min/1.73m^2^) | 104 [87.3;118] | 106 [96.4;120] | 96.0 [69.6;112] | <0.001 |
| Fasting glucose (mmol/l) | 8.52 [7.01;11.9] | 8.24 [6.76;11.1] | 9.28 [7.63;13.0] | 0.068 |
| HbA1c (%) | 9.17 [7.57;11.1] | 8.99 [7.40;10.9] | 9.80 [8.01;11.9] | 0.011 |
| TCH (mmol/L) | 4.58 [3.79;5.53] | 4.52 [3.78;5.27] | 4.80 [3.87;6.02] | 0.074 |
| TG (mmol/L) | 1.37 [0.92;2.14] | 1.33 [0.86;2.01] | 1.63 [1.06;2.42] | 0.023 |
| HDL-C (mmol/L) | 1.19 [1.01;1.37] | 1.19 [1.01;1.38] | 1.15 [0.97;1.37] | 0.482 |
| LDL-C (mmol/L) | 2.74 [2.24;3.43] | 2.67 [2.24;3.38] | 2.95 [2.25;3.54] | 0.084 |
| TCH/HDL-C ratio | 3.76 [3.24;4.65] | 3.69 [3.17;4.50] | 3.95 [3.28;5.05] | 0.021 |
| APOB (g/L) | 0.92 [0.77;1.18] | 0.90 [0.76;1.09] | 1.00 [0.81;1.33] | 0.002 |
| APOA1 (g/L) | 1.22 [1.05;1.41] | 1.25 [1.08;1.42] | 1.17 [1.02;1.34] | 0.018 |
| APOB/APOA1 ratio | 0.77 [0.61;0.97] | 0.73 [0.57;0.93] | 0.91 [0.68;1.13] | <0.001 |
| Lipoprotein a (mg/L) | 198 [91.6;349] | 187 [83.4;312] | 210 [113;444] | 0.032 |
| Hemologbin (g/L) | 142 [126;152] | 143 [130;154] | 137 [118;148] | 0.001 |
| Neutrophils (%) | 59.3 [52.0;66.3] | 57.5 [51.5;63.9] | 62.5 [54.0;76.1] | <0.001 |
| NLR | 2.01 [1.48;3.12] | 1.94 [1.44;2.64] | 2.40 [1.60;4.46] | 0.007 |
| hsCRP (mg/dL) | 1.07 [0.50;6.04] | 1.11 [0.50;6.04] | 1.03 [0.52;28.1] | 0.101 |

^a^Discrete values expressed as number (percentage).

^b^Continuous values expressed as means (SD) if normally distributed or median (interquartile range) if skewed.

Abbreviations: DM, diabetes mellitus; DKD, diabetic kidney disease; BMI, body mass index; RAS, renin-angiotensin system; SGLT-2, sodium-dependent glucose transporters 2; GLP-1RA, glucagon-like peptide-1 receptor agonist; UntOPN, urinary n-terminal osteopontin; UntOCR, urinary n-terminal osteopontin to creatinine ratio; GDF15, growth differentiation factor 15; UGDF15, urinary growth differentiation factor 15; UGCR, urinary growth differentiation factor 15 to creatinine ratio; UACR, urinary albumin to creatinine ratio; TCO_2_, total carbon dioxide; GA, glycosylated albumin; α-HBDH, α-hydroxybutyrate dehydrogenase; ALP, alkaline phosphatase; CK, creatine phosphokinase; BUN, blood urea nitrogen; UA, uric acid; eGFR, estimated glomerular filtration rate; TCH, total cholesterol; TG, triglyceride; HDL-C, high-density lipoprotein cholesterol; LDL-C, low-density lipoprotein cholesterol; APOA1, apolipoprotein A1; APOB, apolipoprotein B; NLR, neutrophil to lymphocyte ratio; hsCRP, high-sensitive C-reactive protein.

Table S3. Baseline characteristics stratified by DKD onset in enrolled 126 DM patients in the prospective cohort.

|  | Total  (N=126) | DM  (N=81) | DKD onset  (N=45) | p.overall |
| --- | --- | --- | --- | --- |
| Sociodemographics: |  |  |  |  |
| Age | 54.5 [46.2;62.0] | 54.0 [47.0;59.0] | 56.0 [44.0;66.0] | 0.268 |
| Female, n (%)^a^ | 45 (35.7%) | 31 (38.3%) | 14 (31.1%) | 0.542 |
| BMI (kg/m^2^)^b^ | 24.8 [22.7;27.3] | 25.0 [22.9;27.8] | 24.2 [22.1;26.6] | 0.254 |
| Duration of diabetes (years) | 6.00 [2.00;11.8] | 5.00 [1.00;10.0] | 10.0 [3.00;18.0] | 0.001 |
| Smoking, n (%) | 44 (34.9%) | 22 (27.2%) | 22 (48.9%) | 0.024 |
| Alcohol, n (%) | 36 (28.6%) | 21 (25.9%) | 15 (33.3%) | 0.499 |
| Comorbid illness |  |  |  |  |
| Hypertension, n (%) | 51 (40.5%) | 31 (38.3%) | 20 (44.4%) | 0.626 |
| Hyperlipemia, n (%) | 61 (48.4%) | 35 (43.2%) | 26 (57.8%) | 0.167 |
| Hyperuricemia, n (%) | 17 (13.5%) | 12 (14.8%) | 5 (11.1%) | 0.756 |
| Medication: |  |  |  |  |
| RAS inhibitor, n (%) | 26 (20.6%) | 17 (21.0%) | 9 (20.0%) | 1.000 |
| SGLT-2 inhibitor, n (%) | 45 (35.7%) | 24 (29.6%) | 21 (46.7%) | 0.086 |
| GLP-1RA, n (%) | 20 (15.9%) | 13 (16.0%) | 7 (15.6%) | 1.000 |
| Metformin, n (%) | 62 (49.2%) | 41 (50.6%) | 21 (46.7%) | 0.811 |
| Laboratory data |  |  |  |  |
| UntOPN (ng/ml) | 39.9 [23.8;101] | 31.6 [23.3;54.3] | 85.4 [30.5;250] | <0.001 |
| UntOCR (μg/mmol) | 4.46 [2.61;16.8] | 3.41 [2.45;6.69] | 12.9 [5.69;41.4] | <0.001 |
| Plasma ntOPN (ng/mL) | 167 [120;237] | 176 [131;244] | 157 [111;225] | 0.136 |
| UGDF15 (pg/mL) | 3219 [2135;4160] | 3058 [2007;3988] | 3272 [2441;4239] | 0.280 |
| UGCR (ng/mmol) | 380 [265;553] | 345 [239;429] | 560 [347;774] | <0.001 |
| Plasma GDF15 (pg/mL) | 165 [113;297] | 157 [112;242] | 234 [123;427] | 0.066 |
| UACR (mg/g) | 13.6 [9.0;20.0] | 11.7 [7.3;14.9] | 20.4 [16.0;24.0] | <0.001 |
| TCO2 (mmol/L) | 26.0 [23.9;27.4] | 26.0 [24.0;27.3] | 25.8 [23.9;27.9] | 0.807 |
| Albumin (g/L) | 43.5 [40.7;45.9] | 43.6 [41.5;45.9] | 43.5 [39.8;46.1] | 0.695 |
| Globulin (g/L) | 25.6 [22.4;28.3] | 25.3 [22.3;28.1] | 25.6 [22.8;28.3] | 0.746 |
| Pre-albumin (mg/L) | 242 [192;276] | 243 [206;277] | 228 [184;270] | 0.186 |
| GA% | 17.7 [14.0;24.6] | 18.1 [14.1;24.5] | 17.7 [13.9;24.6] | 0.955 |
| α-HBDH (U/L) | 140 [123;156] | 137 [123;152] | 141 [119;165] | 0.445 |
| ALP (U/L) | 86.9 [71.4;107] | 87.0 [69.0;108] | 86.9 [73.0;99.0] | 0.823 |
| CK (U/L) | 76.5 [54.2;101] | 71.0 [48.0;95.0] | 85.0 [59.0;133] | 0.062 |
| BUN (mmol/L) | 5.86 [4.90;7.00] | 5.84 [4.89;6.81] | 5.96 [5.02;7.19] | 0.341 |
| Cystatin C (mg/L) | 0.88 [0.78;1.01] | 0.88 [0.77;0.98] | 0.88 [0.80;1.05] | 0.440 |
| UA (μmol/L) | 286 [242;360] | 298 [247;366] | 273 [234;348] | 0.172 |
| Creatinine (μmol/L) | 46.3 [38.2;54.8] | 47.0 [39.8;55.5] | 43.8 [36.8;53.8] | 0.132 |
| eGFR_cr_ (ml/min/1.73m2) | 117 [108;128] | 117 [109;126] | 118 [103;133] | 0.467 |
| eGFR_cr-cys_(ml/min/1.73m2) | 105 [93.6;120] | 105 [96.8;119] | 112 [84.2;122] | 0.825 |
| Fasting glucose (mmol/l) | 8.26 [7.07;11.0] | 8.44 [7.15;10.9] | 8.12 [6.76;11.5] | 0.937 |
| HbA1c (%) | 8.91 [7.48;11.0] | 8.93 [8.10;10.8] | 8.50 [7.34;11.4] | 0.897 |
| TCH (mmol/L) | 4.54 [3.76;5.20] | 4.48 [3.76;5.11] | 4.57 [3.78;5.57] | 0.390 |
| TG (mmol/L) | 1.29 [0.91;2.02] | 1.29 [0.91;1.96] | 1.30 [0.92;2.30] | 0.735 |
| HDL-C (mmol/L) | 1.17 [1.00;1.37] | 1.13 [0.99;1.33] | 1.25 [1.02;1.42] | 0.257 |
| LDL-C (mmol/L) | 2.67 [2.26;3.20] | 2.72 [2.31;3.21] | 2.65 [2.19;3.10] | 0.704 |
| TCH/HDL-C ratio | 3.65 [3.25;4.50] | 3.69 [3.18;4.47] | 3.59 [3.29;4.55] | 0.839 |
| APOB (g/L) | 0.90 [0.76;1.07] | 0.90 [0.76;1.07] | 0.88 [0.76;1.13] | 0.897 |
| APOA1 (g/L) | 1.22 [1.05;1.41] | 1.22 [1.05;1.41] | 1.25 [1.06;1.42] | 0.963 |
| APOB/APOA1 ratio | 0.74 [0.59;0.94] | 0.72 [0.60;0.93] | 0.79 [0.59;0.97] | 0.649 |
| Lipoprotein a (mg/L) | 198 [77.8;302] | 207 [81.0;312] | 157 [77.0;282] | 0.444 |
| Hemologbin (g/L) | 144 [130;153] | 145 [133;154] | 142 [129;149] | 0.174 |
| Neutrophils (%) | 57.7 [51.5;64.2] | 57.5 [50.9;62.4] | 59.5 [52.3;69.8] | 0.119 |
| NLR | 1.93 [1.42;2.70] | 1.93 [1.43;2.53] | 1.95 [1.42;4.04] | 0.470 |
| hsCRP (mg/dL) | 1.71 [0.50;6.87] | 1.40 [0.50;6.04] | 2.20 [0.51;7.53] | 0.425 |

^a^Discrete values expressed as number (percentage).

^b^Continuous values expressed as means (SD) if normally distributed or median (interquartile range) if skewed.

Abbreviations: DM, diabetes mellitus; DKD, diabetic kidney disease; BMI, body mass index; RAS, renin-angiotensin system; SGLT-2, sodium-dependent glucose transporters 2; GLP-1RA, glucagon-like peptide-1 receptor agonist; UntOPN, urinary n-terminal osteopontin; UntOCR, urinary n-terminal osteopontin to creatinine ratio; GDF15, growth differentiation factor 15; UGDF15, urinary growth differentiation factor 15; UGCR, urinary growth differentiation factor 15 to creatinine ratio; UACR, urinary albumin to creatinine ratio; TCO2, total carbon dioxide; GA, glycosylated albumin; α-HBDH, α-hydroxybutyrate dehydrogenase; ALP, alkaline phosphatase; CK, creatine phosphokinase; BUN, blood urea nitrogen; UA, uric acid; eGFR, estimated glomerular filtration rate; TCH, total cholesterol; TG, triglyceride; HDL-C, high-density lipoprotein cholesterol; LDL-C, low-density lipoprotein cholesterol; APOA1, apolipoprotein A1; APOB, apolipoprotein B; NLR, neutrophil to lymphocyte ratio; hsCRP, high-sensitive C-reactive protein.

Table S4. Baseline characteristics stratified by DKD progression in enrolled 126 DM patients in the prospective cohort.

|  | Total  (N=126) | DM  (N=113) | DKD progression  (N=13) | p.overall |
| --- | --- | --- | --- | --- |
| Sociodemographics: |  |  |  |  |
| Age | 54.5 [46.2;62.0] | 55.0 [47.0;62.0] | 54.0 [40.0;61.0] | 0.721 |
| Female, n (%): | 45 (35.7%) | 42 (37.2%) | 3 (23.1%) | 0.376 |
| BMI (kg/m2) | 24.8 [22.7;27.3] | 24.4 [22.7;27.6] | 26.3 [22.1;27.1] | 0.892 |
| Duration of diabetes (years) | 6.00 [2.00;11.8] | 6.00 [2.00;10.0] | 10.0 [5.00;19.0] | 0.093 |
| Smoking, n (%): | 44 (34.9%) | 37 (32.7%) | 7 (53.8%) | 0.217 |
| Alcohol, n (%): | 36 (28.6%) | 32 (28.3%) | 4 (30.8%) | 1.000 |
| Comorbid illnesses: |  |  |  |  |
| Hypertension, n (%): | 51 (40.5%) | 44 (38.9%) | 7 (53.8%) | 0.460 |
| Hyperlipemia, n (%): | 61 (48.4%) | 52 (46.0%) | 9 (69.2%) | 0.196 |
| Hyperuricemia, n (%): | 17 (13.5%) | 14 (12.4%) | 3 (23.1%) | 0.382 |
| Medication: |  |  |  |  |
| RAS inhibitor, n (%): | 26 (20.6%) | 23 (20.4%) | 3 (23.1%) | 0.730 |
| SGLT-2 inhibitor, n (%): | 45 (35.7%) | 40 (35.4%) | 5 (38.5%) | 1.000 |
| GLP-1RA, n (%): | 20 (15.9%) | 17 (15.0%) | 3 (23.1%) | 0.433 |
| Metformin, n (%): | 62 (49.2%) | 54 (47.8%) | 8 (61.5%) | 0.518 |
| Laboratory data: |  |  |  |  |
| UntOPN (ng/ml) | 39.9 [23.8;101] | 34.6 [23.5;81.9] | 110 [76.0;330] | <0.001 |
| UntOCR (μg/mmol) | 4.46 [2.61;16.8] | 3.75 [2.51;12.9] | 30.2 [11.6;60.6] | <0.001 |
| Plasma ntOPN (ng/mL) | 167 [120;237] | 165 [118;239] | 180 [137;228] | 0.622 |
| UGDF-15 (pg/mL) | 3219 [2135;4160] | 3211 [2007;4100] | 3907 [2490;4754] | 0.135 |
| UGCR (ng/mmol) | 380 [265;553] | 354 [257;479] | 717 [499;795] | <0.001 |
| Plasma GDF-15 (pg/mL) | 165 [113;297] | 165 [113;276] | 164 [94.0;361] | 0.829 |
| UACR (mg/g) | 13.6 [9.0;20.0] | 13.2 [8.4;18.7] | 23.0 [16.7;24.3] | <0.001 |
| TCO2 (mmol/L) | 26.0 [23.9;27.4] | 26.0 [24.0;27.6] | 24.8 [23.2;26.6] | 0.188 |
| Albumin (g/L) | 43.5 [40.7;45.9] | 43.6 [40.8;45.7] | 42.7 [39.8;47.7] | 0.639 |
| Globulin (g/L) | 25.6 [22.4;28.3] | 25.6 [22.5;28.5] | 24.9 [21.4;27.2] | 0.478 |
| Pre-albumin (mg/L) | 242 [192;276] | 236 [197;273] | 256 [142;284] | 0.825 |
| GA% | 17.7 [14.0;24.6] | 17.7 [13.8;24.6] | 17.7 [15.1;23.5] | 0.694 |
| α-HBDH (U/L) | 140 [123;156] | 140 [123;155] | 128 [110;174] | 0.594 |
| ALP (U/L) | 86.9 [71.4;107] | 86.0 [70.8;108] | 92.0 [81.0;99.0] | 0.348 |
| CK (U/L) | 76.5 [54.2;101] | 75.0 [53.0;95.0] | 90.0 [55.0;136] | 0.229 |
| BUN (mmol/L) | 5.86 [4.90;7.00] | 5.96 [4.91;6.93] | 5.58 [4.54;7.16] | 0.770 |
| Cystatin C (mg/L) | 0.88 [0.78;1.01] | 0.88 [0.78;0.99] | 0.82 [0.72;1.01] | 0.483 |
| UA (μmol/L) | 286 [242;360] | 291 [241;361] | 279 [258;348] | 0.873 |
| Creatinine (μmol/L) | 46.3 [38.2;54.8] | 46.9 [39.6;55.1] | 38.6 [30.0;47.3] | 0.024 |
| eGFR_cr_ (ml/min/1.73m2) | 117 [108;128] | 117 [108;128] | 139 [108;146] | 0.039 |
| eGFR_cr-cys_(ml/min/1.73m2) | 105 [93.6;120] | 105 [93.6;119] | 117 [86.8;142] | 0.182 |
| Fasting glucose (mmol/l) | 8.26 [7.07;11.0] | 8.19 [7.06;10.9] | 10.5 [7.86;13.2] | 0.090 |
| HbA1c (%) | 8.91 [7.48;11.0] | 8.93 [7.50;11.0] | 8.70 [7.20;10.9] | 0.709 |
| TCH (mmol/L) | 4.54 [3.76;5.20] | 4.50 [3.77;5.13] | 5.03 [3.28;5.82] | 0.485 |
| TG (mmol/L) | 1.29 [0.91;2.02] | 1.29 [0.91;1.96] | 1.32 [0.82;3.16] | 0.365 |
| HDL-C (mmol/L) | 1.17 [1.00;1.37] | 1.18 [1.00;1.37] | 1.13 [0.93;1.31] | 0.727 |
| LDL-C (mmol/L) | 2.67 [2.26;3.20] | 2.67 [2.31;3.13] | 2.95 [2.01;3.53] | 0.645 |
| TCH/HDL-C ratio | 3.65 [3.25;4.50] | 3.59 [3.25;4.45] | 4.30 [3.53;5.34] | 0.191 |
| APOB (g/L) | 0.90 [0.76;1.07] | 0.89 [0.76;1.07] | 1.07 [0.87;1.19] | 0.080 |
| APOA1 (g/L) | 1.22 [1.05;1.41] | 1.23 [1.06;1.41] | 1.14 [1.03;1.41] | 0.356 |
| APOB/APOA1 ratio | 0.74 [0.59;0.94] | 0.72 [0.58;0.92] | 0.96 [0.70;1.23] | 0.022 |
| Lipoprotein a (mg/L) | 198 [77.8;302] | 203 [80.0;310] | 156 [69.0;271] | 0.453 |
| Hemologbin (g/L) | 144 [130;153] | 145 [130;154] | 142 [136;147] | 0.518 |
| Neutrophils (%) | 57.7 [51.5;64.2] | 57.0 [51.2;63.6] | 64.0 [60.9;72.1] | 0.013 |
| NLR | 1.93 [1.42;2.70] | 1.92 [1.42;2.66] | 2.13 [1.52;2.81] | 0.432 |
| hsCRP (mg/dL) | 1.71 [0.50;6.87] | 1.66 [0.50;7.15] | 3.38 [0.50;6.04] | 0.994 |

^a^Discrete values expressed as number (percentage).

^b^Continuous values expressed as means (SD) if normally distributed or median (interquartile range) if skewed.

Abbreviations: DM, diabetes mellitus; DKD, diabetic kidney disease; BMI, body mass index; RAS, renin-angiotensin system; SGLT-2, sodium-dependent glucose transporters 2; GLP-1RA, glucagon-like peptide-1 receptor agonist; UntOPN, urinary n-terminal osteopontin; UntOCR, urinary n-terminal osteopontin to creatinine ratio; GDF15, growth differentiation factor 15; UGDF15, urinary growth differentiation factor 15; UGCR, urinary growth differentiation factor 15 to creatinine ratio; UACR, urinary albumin to creatinine ratio; TCO2, total carbon dioxide; GA, glycosylated albumin; α-HBDH, α-hydroxybutyrate dehydrogenase; ALP, alkaline phosphatase; CK, creatine phosphokinase; BUN, blood urea nitrogen; UA, uric acid; eGFR, estimated glomerular filtration rate; TCH, total cholesterol; TG, triglyceride; HDL-C, high-density lipoprotein cholesterol; LDL-C, low-density lipoprotein cholesterol; APOA1, apolipoprotein A1; APOB, apolipoprotein B; NLR, neutrophil to lymphocyte ratio; hsCRP, high-sensitive C-reactive protein.

Table S5. Spearman correlation coefficients among the novel biomarkers and DKD diagnosis in the cross-sectional cohort.

|  | DKD diagnosis | UntOCR | Plasma ntOPN | UGCR | Plasma GDF15 | UACR | eGFR_cr-cys_ |
| --- | --- | --- | --- | --- | --- | --- | --- |
| DKD diagnosis |  | 0.2695 | 0.0659 | 0.2654 | 0.2706 | 0.7618 | -0.2489 |
| UntOCR | <0.001 |  | 0.1487 | 0.6732 | 0.3204 | 0.4108 | -0.0505 |
| Plasma ntOPN | 0.2431 | 0.0081 |  | 0.0925 | 0.1705 | 0.0712 | -0.0712 |
| UGCR | <0.001 | <0.001 | 0.1007 |  | 0.4135 | 0.3476 | -0.1618 |
| Plasma GDF15 | <0.001 | <0.001 | 0.0024 | <0.001 |  | 0.3746 | -0.5473 |
| UACR | <0.001 | <0.001 | 0.2067 | <0.001 | <0.001 |  | -0.266 |
| eGFR_cr-cys_ | <0.001 | 0.3707 | 0.207 | 0.0039 | <0.001 | <0.001 |  |

Spearman correlation coefficients in the top-right diagonal and their P values in the bottom-left diagonal.

Table S6. Spearman correlation coefficients among the novel biomarkers and clinical outcomes in the prospective cohort.

|  | DKD occurrence | UntOCR | Plasma ntOPN | UGCR | Plasma GDF15 | UACR | eGFR_cr-cys_ | DKD progression |
| --- | --- | --- | --- | --- | --- | --- | --- | --- |
| DKD occurrence |  | 0.4406 | -0.1332 | 0.3948 | 0.1642 | 0.5189 | -0.0198 | 0.4551 |
| UntOCR | <0.001 |  | -0.1127 | 0.7125 | 0.2829 | 0.4956 | 0.1146 | 0.3640 |
| Plasma ntOPN | 0.137 | 0.2088 |  | -0.084 | 0.0341 | -0.0417 | -0.0891 | 0.0441 |
| UGCR | <0.001 | <0.001 | 0.3496 |  | 0.3549 | 0.3824 | -0.0838 | 0.3199 |
| Plasma GDF15 | 0.0662 | 0.0013 | 0.7046 | <0.001 |  | 0.2385 | -0.5026 | -0.0194 |
| UACR | <0.001 | <0.001 | 0.643 | <0.001 | 0.0072 |  | -0.0358 | 0.3415 |
| eGFR_cr-cys_ | 0.8257 | 0.2013 | 0.3213 | 0.3508 | <0.001 | 0.6904 |  | 0.1194 |
| DKD progression | <0.001 | <0.001 | 0.6238 | <0.001 | 0.8296 | <0.001 | 0.1828 |  |

Spearman correlation coefficients in the top-right diagonal and their P values in the bottom-left diagonal.

Abbreviations: DKD, diabetic kidney disease; UntOCR, urinary n-terminal osteopontin to creatinine ratio; ntOPN, n-terminal osteopontin; UGCR, urinary growth differentiation factor 15 to creatinine ratio; GDF15, growth differentiation factor 15; UACR, urinary albumin to creatinine ratio; eGFR, estimated glomerular filtration rate.

Table S7. The IDI and NRI for models in Figure 4 and Figure S6.

|  | DKD occurrence | | | | DKD progression | | | |
| --- | --- | --- | --- | --- | --- | --- | --- | --- |
|  | Est. | Lower | Upper | p-value | Est. | Lower | Upper | p-value |
| **UGCR+UntOCR+ eGFR_cr-cys_ +UACR vs. eGFR_cr-cys_ +UACR** | | | | | | | | |
| IDI | 0.0467 | 0.0065 | 0.0869 | 0.0229 | 0.0624 | -0.0181 | 0.1428 | 0.1286 |
| NRI | 0.5580 | 0.2069 | 0.9092 | 0.0018 | 0.7297 | 0.199 | 1.2605 | 0.0070 |
| **UGCR+UntOCR+ eGFR_cr_ +UACR vs. eGFR_cr_ +UACR** | | | | | | | | |
| IDI | 0.043 | 0.0043 | 0.0817 | 0.0295 | 0.0341 | -0.0365 | 0.1048 | 0.3436 |
| NRI | 0.464 | 0.1089 | 0.8195 | 0.0104 | 0.3989 | -0.1604 | 0.9582 | 0.1621 |
| **UGCR+plasma GDF15+eGFR_cr-cys_+UACR vs. eGFR_cr-cys_+UACR** | | | | | | | | |
| IDI | 0.0478 | 0.0072 | 0.0883 | 0.0209 | 0.0607 | -0.0169 | 0.1384 | 0.1254 |
| NRI | 0.6025 | 0.2537 | 0.9512 | 0.0007 | 0.7651 | 0.2352 | 1.2951 | 0.0047 |
| **UntOCR+plasma ntOPN+eGFR_cr-cys_+UACR vs. eGFR_cr-cys_+UACR** | | | | | | | | |
| IDI | 0.0359 | 7e-04 | 0.0711 | 0.0455 | 0.0463 | -0.0299 | 0.1225 | 0.2338 |
| NRI | 0.4741 | 0.1219 | 0.8263 | 0.0083 | 0.2097 | -0.3623 | 0.7816 | 0.4725 |
| **UGCR+plasma GDF15+eGFR_cr_+UACR vs. eGFR_cr_+UACR** | | | | | | | | |
| IDI | 0.0423 | 0.0043 | 0.0803 | 0.0291 | 0.0377 | -0.0332 | 0.1087 | 0.2968 |
| NRI | 0.5778 | 0.2279 | 0.9277 | 0.0012 | 0.2628 | -0.3087 | 0.8342 | 0.3675 |
| **UntOCR+plasma ntOPN+eGFR_cr_+UACR vs. eGFR_cr_+UACR** | | | | | | | | |
| IDI | 0.0359 | 4e-04 | 0.0713 | 0.0475 | 0.0257 | -0.0387 | 0.0901 | 0.4347 |
| NRI | 0.4346 | 0.0785 | 0.7906 | 0.0168 | 0.0531 | 0.005 | 0.1012 | 0.0304 |

Note: All multivariate logistics models were adjusted by age, gender, BMI. Lower and Upper of 95% confidence interval are described.

Abbreviations: IDI, integrated discrimination improvement; NRI, net reclassification index; DKD, diabetic kidney disease; UGCR, urinary growth differentiation factor 15 to creatinine ratio; GDF15, growth differentiation factor 15; UntOCR, urinary n-terminal osteopontin to creatinine ratio; ntOPN, n-terminal osteopontin; eGFR, estimated glomerular filtration rate; UACR, urinary albumin to creatinine ratio; BMI, body mass index.


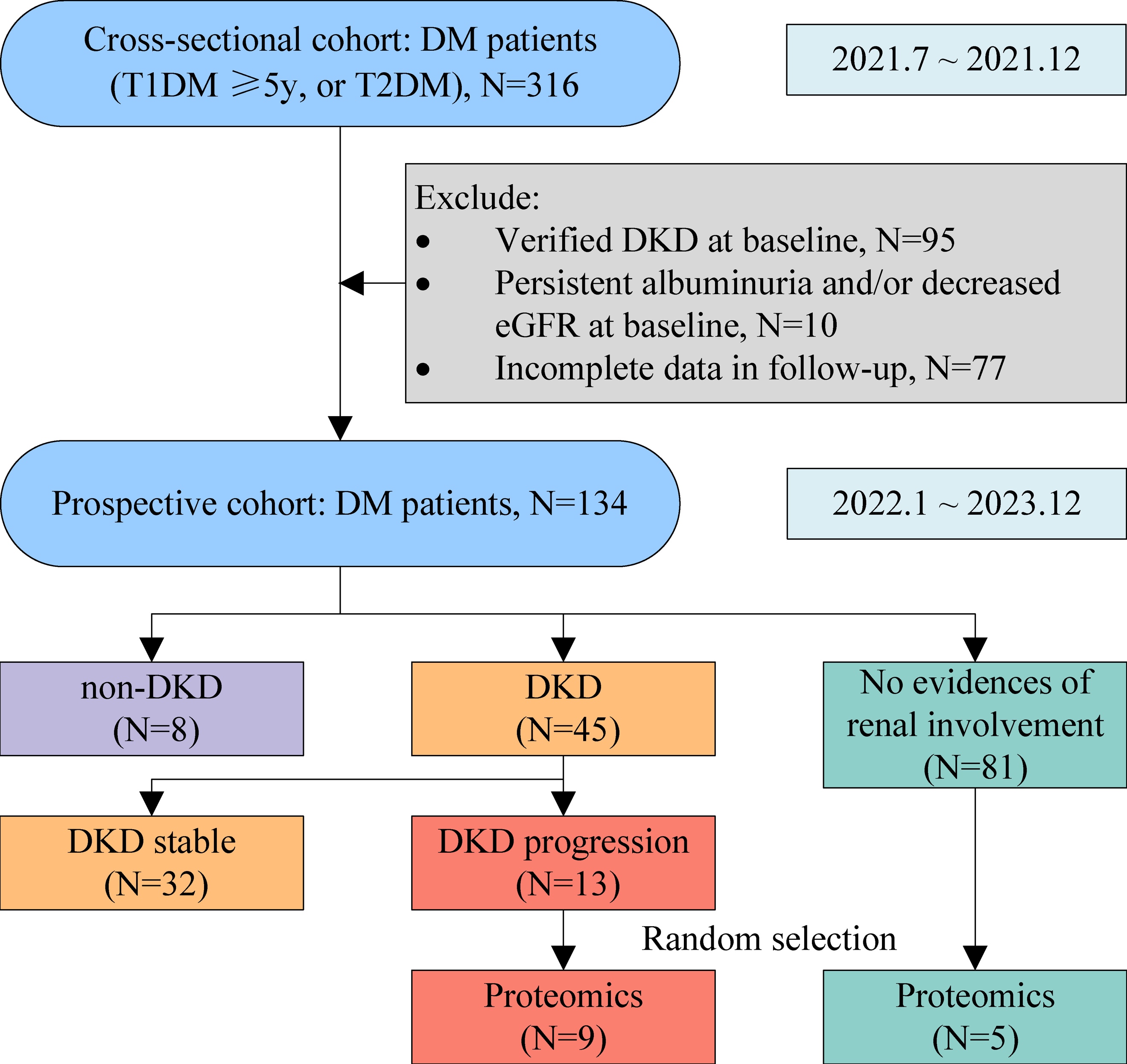


Figure S1. Overview of study design and cohort participants. In the prospective cohort phase, a total of 134 DM patients without kidney injury were included and followed. Because of the high cost of SOMAscan measurements, we randomly selected baseline plasma samples from 9 patients with DKD progression (red square), and 5 patients without renal involvement (green square) during the follow-up period for further proteomics assays. DM, diabetes mellitus; T1DM, Type 1 diabetes mellitus; T2DM, Type 2 diabetes mellitus; DKD, diabetic kidney disease; non-DKD, non-diabetic kidney diseases; eGFR, estimated glomerular filtration rate.


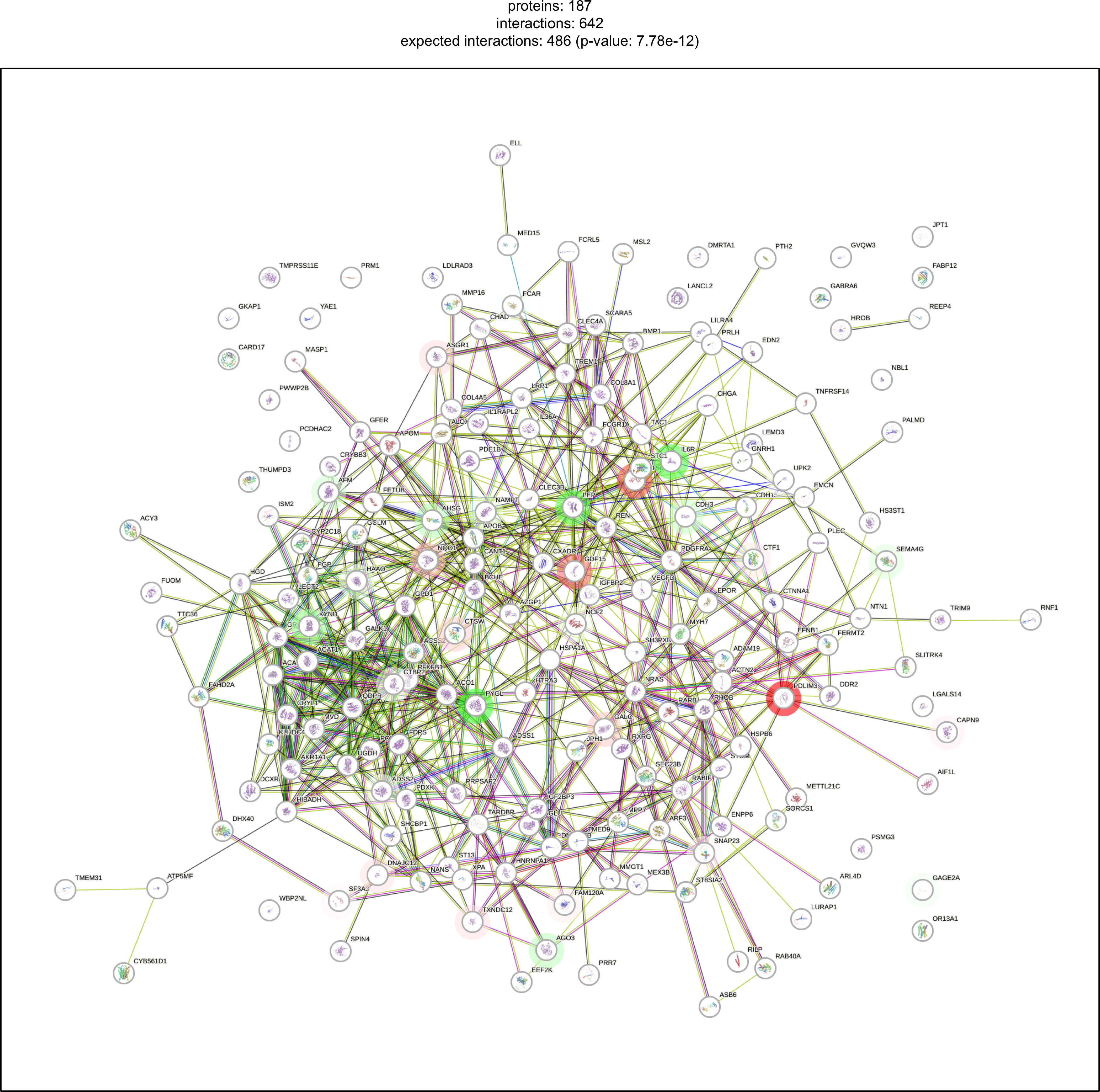


Figure S2. Protein-protein interaction (PPI) network showing the interactions of differentially expressed proteins based on STRING database. The darker the node, the more core the interaction.


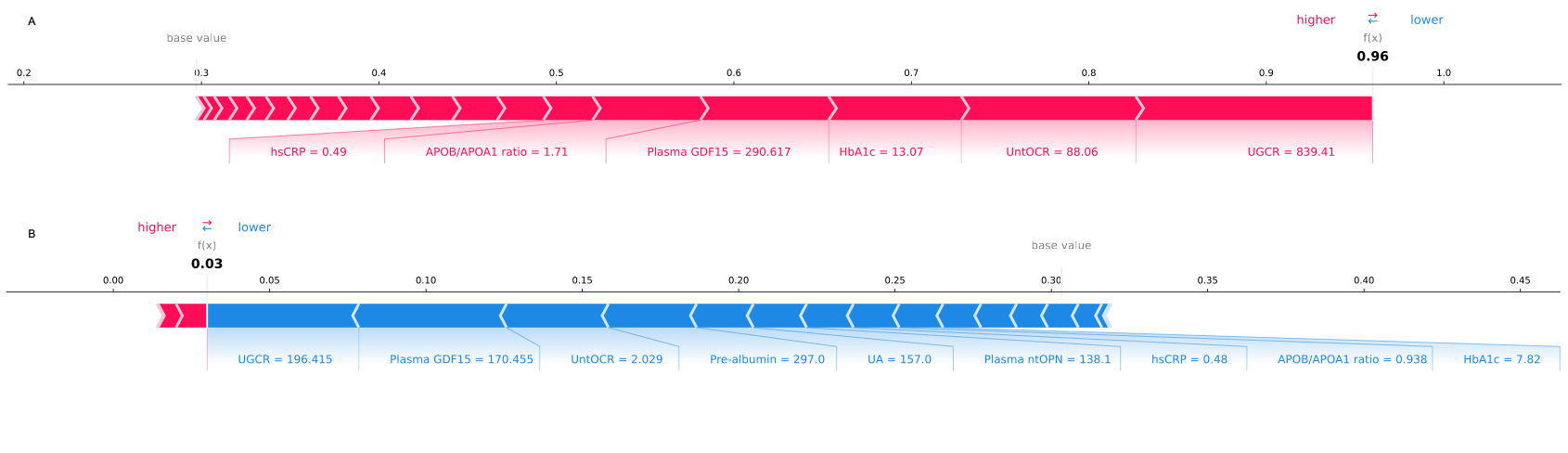


Figure S3. Individuals with a high SHAP value (A) and a low SHAP value (B) for DKD diagnosis in the cross-sectional cohort.


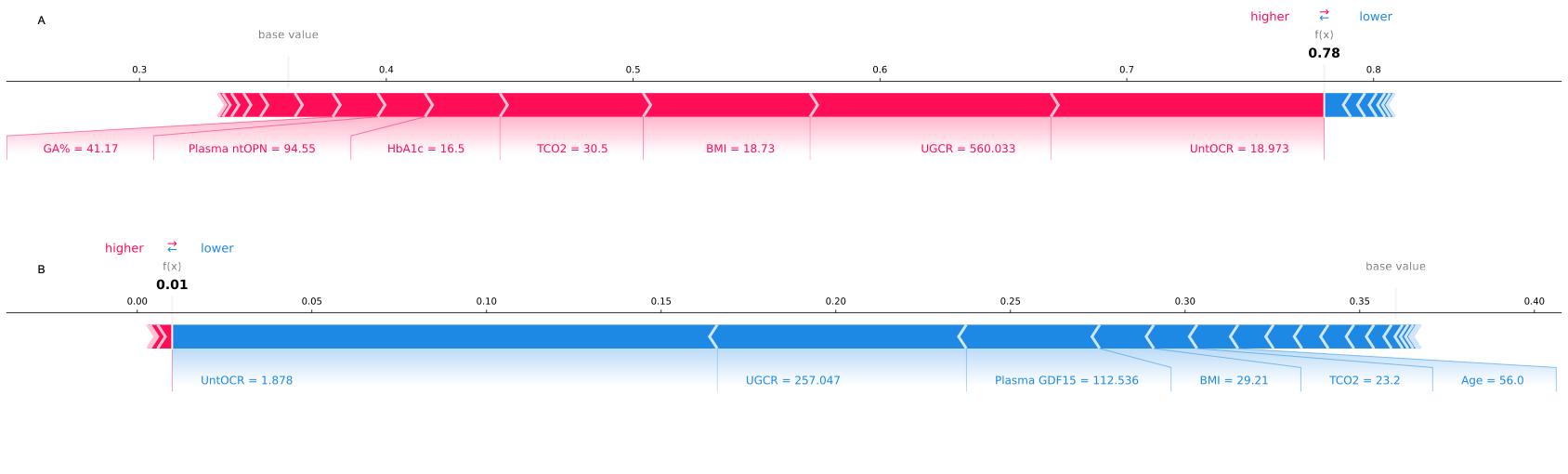


Figure S4. Individuals with a high SHAP value (A) and a low SHAP value (B) for DKD occurrence in the prospective cohort.


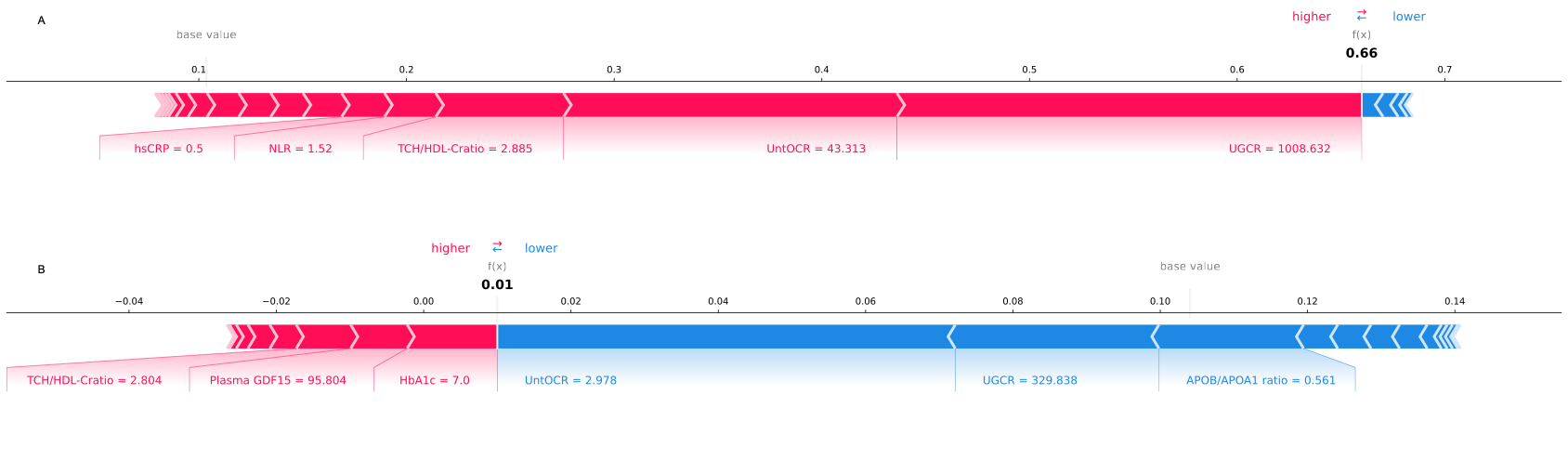


Figure S5. Individuals with a high SHAP value (A) and a low SHAP value (B) for DKD progression in the prospective cohort.


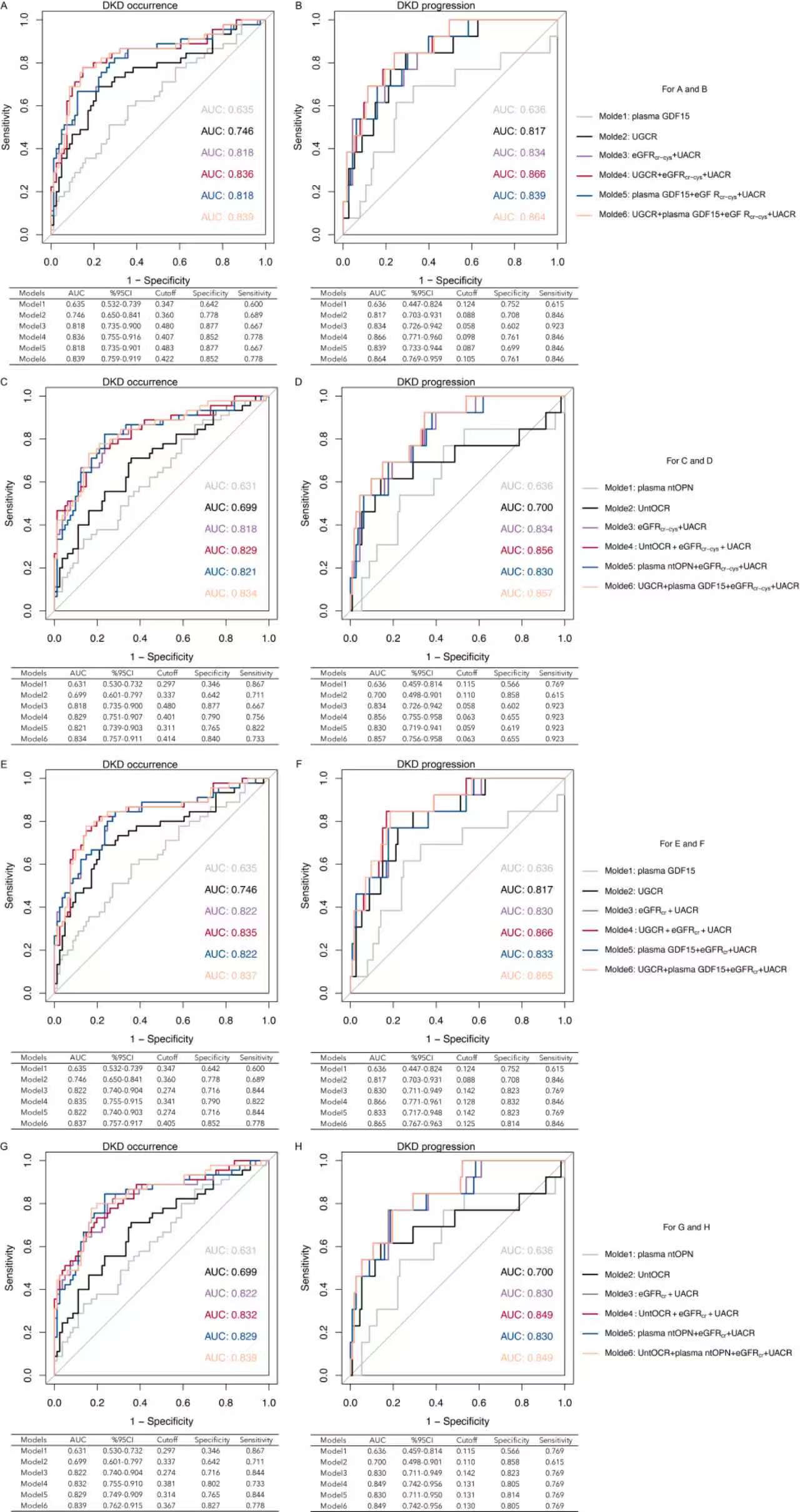


Figure S6. The receiver operator characteristic (ROC) curves of models for DKD prediction, adjusted by age, gender, and BMI. The AUC and its 95% CI, cut-off value, specificity, and sensitivity were listed below the ROC curves for each model. DKD, diabetic kidney disease; BMI, body mass index; GDF15, growth differentiation factor 15; UGCR, urinary growth differentiation factor 15 to creatinine ratio; ntOPN, n-terminal osteopontin; UntOCR, urinary n-terminal osteopontin to creatinine ratio; eGFR, estimated glomerular filtration rate; UACR, urinary albumin to creatinine ratio; AUC, area under the curve; CI, confidence interval.
